# The Impact of Treatment on Quality of Life in Patients with Head and Neck Cancer

**Published:** 2019-01

**Authors:** Marta DĄBROWSKA-BENDER, Robert SŁONIEWSKI, Urszula RELIGIONI, Anna SŁONIEWSKA, Magdalena MILEWSKA, Anna KUPIECKA, Adrianna SOBOL, Anna STANISZEWSKA

**Affiliations:** 1. Dept. of Clinical Dietetics, Medical University of Warsaw, Warsaw, Poland; 2. “Fundation Onko Cafe- Together Better”, Warsaw, Poland; 3. Dept. of Public Health, Medical University of Warsaw, Warsaw, Poland; 4. Collegium of Business Administration, Warsaw School of Economics, Warsaw, Poland; 5. Masovian Oncological Hospital, Wieliszew, Poland; 6. Dept. of Oncological Prevention, Medical University of Warsaw, Warsaw, Poland; 7. Dept. of Experimental and Clinical Pharmacology, Medical University of Warsaw, Warsaw, Poland

## Dear Editor-in-Chief

Every year in the world there are 600,000 new cases of patients diagnosed with head and neck cancer (1,2). In Poland head and neck cancer accounts for 4.5% of all cancer cases which in 2008 reached 6046 (3). The aim of study was to assess the quality of life in patients’ treatment due to head and/or neck cancer. Study was conducted of 48 patients (52.08% men), treatment at the Oncology Center, Maria Skłodowska-Curie Institute in Warsaw, Poland, in 2015. The standardized questionnaire EORTC QLQ-C30 and QLQH&N35 module for these patients.

The study was approved by the local Ethics Committee and the management of the hospital. All subjects gave their written informed consent to participation in the study.

The mean age was 56.29±6.94 yr (range: 43–67 yr). An equal number of respondents had location of tumour: larynx and laryngopharynx and oral cavity (43.75% each) and oropharynx (12.50%). Patients was treatment: radiotherapy (54.17%); chemotherapy (4.17%); surgical treatment (2.08%); radiotherapy and chemotherapy (33.33%); surgery, radiotherapy and chemotherapy (4.17%); surgery and radiotherapy (2.08%). The mean value of subjective assessment of health condition reached 4.04±1.09 and quality of life 3.79±1.17. The statistical analysis did not reveal correlations between subjective assessment of health condition and level of quality of life and sex, age, location of tumour, stage, and type of treatment.

Each of aspects of physical function (difficulties in performing wearisome activities, fatigue during long and short walks, help required in the performance of everyday activities, limitations in the performance of everyday activities, limitations in pursuing hobbies, need for rest during the day and the sense of fatigue) was correlated with sex, age, location of tumour, stage and type of treatment. Physical function was affected solely by the stage and type of treatment. Thus, sex, age, location of tumour did not determine the physical ability of patients.

The sense of pain (dyspnoea, weakness and pain that made the performance of everyday activities more difficult) was not determined by variables: sex, age, location of tumour, stage and type of treatment.

16.7% of patients` health condition did not affect their family life, with 29.2% claimed there was no connection between their health condition and social life. Senior patients experienced significant or very significant difficulties (*P*=0.01).

The results of QLQ-N&H 35 questionnaire are illustrated in Table 1.

**Table 1:** The results of QLQ-H&N35 questionnaire

***QLQ-H&N35***	***N (%)***			
	***None***	***Slight***	***Significant***	***Very significant***
Pain in the mouth	10 (20.8)	19 (39.6)	11 (22.9)	8 (16.7)
Pain in the maxilla	13 (27.1)	19 (39.6)	10 (20.8)	6 (12.5)
Oral sensitivity	4 (8.4)	22 (45.8)	12 (25.0)	10 (20.8)
Sore throat	4 (8.4)	18 (37.5)	16 (33.3)	10 (20.8)
Difficulties in swallowing of liquids	7 (14.6)	17 (35.4)	18 (37.5)	6 (12.5)
Difficulties in swallowing of mashed foods	8 (16.7)	20 (41.7)	11 (22.9)	9 (18.7)
Difficulties in swallowing of solid foods	3 (6.3)	16 (33.3)	18 (37.5)	11 (22.9)
Choking when swallowing	13 (27.1)	12 (25.0)	13 (27.1)	10 (20.8)
Teeth problems	19 (39.6)	12(25.0)	12 (25.0)	5 (10.4)
Difficulties in mouth opening	10 (20.8)	18 (37.5)	17 (35.4)	3 (6.3)
Dry mouth	1 (2.1)	19 (39.6)	22 (45.8)	6 (12.5)
Gluey saliva	4 (8.3)	14 (29.2)	23 (47.9)	7 (14.6)
Impaired smell	7 (14.6)	22 (45.8)	12 (25.0)	7 (14.6)
Impaired taste	2 (4.2)	15 (31.3)	18 (37.5)	13 (27.1)
Cough	10 (20.8)	18 (37.5)	11 (22.9)	9 (18.8)
Hoarse voice	10 (20.8)	17 (35.4)	12 (25.0)	9 (18.8)
Feeling sick	6 (12.5)	17 (35.4)	17 (35.4)	8 (16.7)
Preoccupied with appearance	11 (22.9)	13 (27.1)	13 (27.1)	11 (22.9)
Difficulties in eating	7 (14.6)	17 (35.4)	13 (27.1)	11 (22.9)
Difficulties in eating with the family	17 (35.4)	10 (20.8)	10 (20.8)	11 (22.9)
Difficulties in eating with other people	17 (35.4)	12 (25.0)	13 (27.1)	6 (12.5)
Difficulties in deriving pleasure from eating	6 (12.5)	19 (39.6)	14 (29.2)	9 (18.8)
Difficulties in speaking with other people	12 (25.0)	17 (35.4)	11 (22.9)	8 (16.7)
Difficulties in phone conversations	8 (16.7)	20 (41.7)	10 (20.8)	10 (20.8)
Difficulties in family communication	19 (39.6)	12 (25.0)	12 (25.0)	5 (10.4)
Difficulties in social relations	16 (33.3)	16 (33.3)	11 (22.9)	5 (10.4)
Difficulties when leaving someone else’s house	17 (35.4)	14 (29.2)	9 (18.8)	8 (16.7)
Difficulties in contacts with family and friends	15 (31.3)	17 (35.4)	12 (25.0)	4 (8.3)
Decreased interest in sex	16 (33.3)	16 (33.3)	13 (27.1)	6 (12.5)
Decreased satisfaction with sex	15 (31.3)	14 (29.2)	12 (27.1)	6 (12.5)

Oral sensitivity was more common among patients completed treatment compared against those who still received treatment (*P*=0.04). Women reported primarily difficulties in swallowing of mashed foods (*P*=0.03) and impaired smell (*P*=0.02). A statistically significant relation was found between the sense of a dry mouth and the stage of treatment (*P*=0.00). The difficulties associated with phone conversations were dependent on the health condition of patients (*P*=0.04). The difficulties in family communication significantly impaired the quality of life of patients (*P*=0.01). No such relation was found in case of social relations. There was a correlation between satisfaction with sex and health condition of patients (*P*=0.03).
